# Damaged Neocortical Perineuronal Nets Due to Experimental Focal Cerebral Ischemia in Mice, Rats and Sheep

**DOI:** 10.3389/fnint.2017.00015

**Published:** 2017-08-15

**Authors:** Wolfgang Härtig, Bianca Mages, Susanne Aleithe, Björn Nitzsche, Stephan Altmann, Henryk Barthel, Martin Krueger, Dominik Michalski

**Affiliations:** ^1^Department of Pathophysiology of Neuroglia, Paul Flechsig Institute for Brain Research, University of Leipzig Leipzig, Germany; ^2^Department of Neurology, University of Leipzig Leipzig, Germany; ^3^Department of Nuclear Medicine, University of Leipzig Leipzig, Germany; ^4^Faculty of Veterinary Medicine, Institute of Anatomy, Histology and Embryology, University of Leipzig Leipzig, Germany; ^5^Institute of Anatomy, University of Leipzig Leipzig, Germany

**Keywords:** cerebral ischemia, stroke, animal model, neurovascular unit, extracellular matrix, aggrecan, WFA

## Abstract

As part of the extracellular matrix (ECM), perineuronal nets (PNs) are polyanionic, chondroitin sulfate proteoglycan (CSPG)-rich coatings of certain neurons, known to be affected in various neural diseases. Although these structures are considered as important parts of the neurovascular unit (NVU), their role during evolution of acute ischemic stroke and subsequent tissue damage is poorly understood and only a few preclinical studies analyzed PNs after acute ischemic stroke. By employing three models of experimental focal cerebral ischemia, this study was focused on histopathological alterations of PNs and concomitant vascular, glial and neuronal changes according to the NVU concept. We analyzed brain tissues obtained 1 day after ischemia onset from: (a) mice after filament-based permanent middle cerebral artery occlusion (pMCAO); (b) rats subjected to thromboembolic MACO; and (c) sheep at 14 days after electrosurgically induced focal cerebral ischemia. Multiple fluorescence labeling was applied to explore simultaneous alterations of NVU and ECM. Serial mouse sections labeled with the net marker *Wisteria floribunda* agglutinin (WFA) displayed largely decomposed and nearly erased PNs in infarcted neocortical areas that were demarcated by up-regulated immunoreactivity for vascular collagen IV (Coll IV). Subsequent semi-quantitative analyses in mice confirmed significantly decreased WFA-staining along the ischemic border zone and a relative decrease in the directly ischemia-affected neocortex. Triple fluorescence labeling throughout the three animal models revealed up-regulated Coll IV and decomposed PNs accompanied by activated astroglia and altered immunoreactivity for parvalbumin, a calcium-binding protein in fast-firing GABAergic neurons which are predominantly surrounded by neocortical PNs. Furthermore, ischemic neocortical areas in rodents simultaneously displayed less intense staining of WFA, aggrecan, the net components neurocan, versican and the cartilage link protein (CRTL) as well as markers in net-bearing neurons such as the potassium channel subunit Kv3.1b and neuronal nuclei (NeuN). In summary, theconsistent observations based on three different stroke models confirmed that PNs are highly sensitive constituents of the NVU along with impaired associated GABAergic neurons. These results suggest that PNs could be promising targets of future stroke treatment, and further studies should address their reorganization and plasticity in both stabilizing the acute stroke as well as supportive effects during the chronic phase of stroke.

## Introduction

Despite enormous efforts in both pre-clinical and clinical research, stroke still represents one of the leading causes of death world-wide (Benjamin et al., 2017). Acute stroke treatment is currently focused on the re-opening of cerebral occluded vessels underlying the ischemic event. In this direction, intravenous application of recombinant tissue plasminogen activator (rtPA, Alteplase) within the first 4.5 h after ischemia onset accounts as a proven therapy (Hacke et al., 2008), recently complemented by local interventions such as mechanical thrombectomy (Berkhemer et al., 2015). Due to contra-indications and the narrow time window, only a minority of patients are eligible for these therapies (Dirks and Dippel, 2013). Importantly, more than 1000 preclinical approaches of neuroprotection failed to be transferred into the clinical setting (O’Collins et al., 2006). Among the reasons for this translational roadblock, the long-lasting neuro-centric focus of stroke research has been discussed (Meairs et al., 2006). To overcome this restriction, numerous studies started to explore ischemic consequences upon other cell types than strictly neurons (Lo, 2008; Moskowitz et al., 2010). In this view, the concept of the “neurovascular unit” (NVU) as outlined by del Zoppo (2009, 2010) comprised ischemia-affected neurons as well as vessels, micro-, astro- and oligodendroglia. Of note, this concept also involves the adjacent extracellular matrix (ECM) which is known to critically impact on a variety of cellular functions (del Zoppo, 2009). Based on the NVU concept, our own histochemical analyses after experimental stroke in mice revealed significant alterations of vascular components (Hawkes et al., 2013; Krueger et al., 2015), microtubule-associated protein tau (Michalski et al., 2016) and vesicular neurotransmitter transporters (Michalski et al., 2013). In a first set of experiments that characterized ischemic consequences to the ECM, we were able to show significant alterations of ECM constituents—i.e., degraded perineuronal nets (PNs)—in the subcortical nucleus reticularis thalami (Härtig et al., 2016).

PNs represent polyanionic lattice-like coatings of neurons (Brückner et al., 1993; Celio and Blümcke, 1994; Oohashi et al., 2015; Sorg et al., 2016). They are composed of chondroitin sulfate proteoglycans (CSPGs) and their glycosaminoglycans, hyaluronic acid and tenascins, produced by various neural cell types (Brückner et al., 1993). The chemical heterogeneity of PNs facilitated multiple detection methods including antibodies directed against different CSPGs such as aggrecan (Lander et al., 1997) and tenascins, but also N-acetylgalactosamine-recognizing lectins such as *Wisteria floribunda* agglutinin (WFA; Härtig et al., 1992) and *Vicia villosa* agglutinin (VVA; Nakagawa et al., 1986; Kosaka and Heizmann, 1989). Classical histological detection methods enabled first descriptions of nets by Camillo Golgi and Ramón y Cajal (as summarized for instance by Brauer et al., 1982; Bignami et al., 1992; Celio et al., 1998; Celio, 1999). Notably, Brauer et al. (1982) introduced the term “PNs” based on Golgi impregnation techniques visualizing these web-like coatings in association with glia. PNs frequently surround neurons containing parvalbumin (Kosaka and Heizmann, 1989; Härtig et al., 1992), known as a marker for fast-firing GABAergic neurons co-expressing the voltage-gated potassium channel Kv3.1b (Härtig et al., 1999).

ECM in general and PNs in particular are of increasing interest as they represent modulators of cellular integrity and neural plasticity in health and disease (Rauch, 2004; Carulli et al., 2010; Dityatev et al., 2010; Kwok et al., 2011; Bartus et al., 2012; Frischknecht et al., 2014; Fawcett, 2015; Sorg et al., 2016). For example, polyanionic PNs were considered as rapid buffering system for cations (Härtig et al., 1999; Morawski et al., 2015), and were reported to have neuroprotective properties against glutamate-induced cell death (Okamoto et al., 1994) and oxidative stress (Morawski et al., 2004; Cabungcal et al., 2013; Suttkus et al., 2016b). With respect to functional consequences, PNs were found to mediate the formation of erasure-resistant persistence of memories (Gogolla et al., 2009; Tsien, 2013).

However, data on the changes in PNs after stroke remain rather limited. Hobohm et al. (2005) found 7 days after experimental stroke induced by invasive electrocoagulative occlusion of the middle cerebral artery in rats that PNs were damaged in the primarily affected neocortex and in the more distant thalamic nuclei. Additionally, Karetko-Sysa et al. (2011) reported the disturbance of PNs after focal photothrombotic stroke. Using a similar animal model, ischemia-induced damage of PNs in rat neocortices was analyzed by Bidmon et al. (1998) and by Wieloch’s research group (Madinier et al., 2014; Quattromani et al., 2017). Notably, as another reason for the translational roadblock the type of ischemia induction, i.e., the widely used artificial stroke models (Young et al., 2007; Sommer, 2017) and the selection of a single stroke model (Fisher et al., 2009) were discussed. The consideration of such aspects is crucial to explore ischemic consequences to the ECM and PNs in relation to the cellular components of the NVU. An improved understanding on these circumstances might allow the development of specific treatment strategies addressing the integrity of the NVU with adjacent ECM components.

Therefore, the present study aimed to address ischemia-related alterations of PNs as well as associated neocortical neuronal, glial and vascular alterations in the models of: (i) filament-based permanent middle cerebral artery occlusion (pMCAO) in mice; (ii) thromboembolic MCAO in rats; and (iii) electrosurgically induced pMCAO in sheep.

## Experimental Procedures

### Study Design and Induction of Focal Cerebral Ischemia

Animal experiments were performed according to the European Union Directive 2010/63/EU and the protocol was approved by the local authority (Regierungspräsidium Leipzig; reference numbers: TVV 51/14 for mice, TVV 34/11 for rats and TVV 56/15 for sheep). The study was carried out with *n* = 5 C57BL/6 mice of about 25 g body weight and *n* = 3 Wistar rats of about 300 g body weight (both breed by Charles River, Sulzfeld, Germany), which were randomly included, and with three male adult sheep from the nucleus breed of the educational manor (Faculty for Veterinary Medicine, University of Leipzig).

Focal cerebral ischemia in mice was induced by a right-sided pMCAO using a filament-based approach as originally described by Longa et al. (1989) with some minor modifications. Briefly, a standardized silicon-coated 6-0 monofilament (Doccol Corporation, Redlands, CA, USA) was inserted into the right external carotid artery and then moved forward into the internal carotid artery until bending was observed or resistance was felt. Mice were anesthetized with etomidate (33 mg/kg body weight i.p.; Hypnomidate, Janssen-Cilag, Neuss, Germany), added by local subcutaneous injections of lidocaine (Xylocitin 1%, mibe, Brehna, Germany) at the area of surgery. In parallel, the body temperature was monitored and adjusted to 37°C by a thermostatically controlled warming pad via a rectal probe (Fine Science Tools, Heidelberg, Germany). After surgery, mice were placed on a commercially available warming pad (37°C) until recovery from anesthesia.

In rats, focal cerebral ischemia was realized by applying a thromboembolic model as originally described by Zhang et al. (1997) with some minor modifications. Briefly, after surgical exposure of right-sided cervical arteries, a polyethylene (PE) tube with tapered end was introduced into the external carotid artery and moved forward through the internal carotid artery up to the origin of the middle cerebral artery (approximately 16 mm from carotid bifurcation). Blood was collected in a PE tube and allowed to clot on a warming pad (37°C) for 2 h followed by overnight storage at 4°C before surgery. Individual blood clots with a length of 45 mm were prepared and injected with a small volume of saline. Finally, the catheter was removed. During surgical procedure, rats were anesthetized using 2.0%–2.5% isoflurane (Isofluran Baxter; Baxter, Unterschleißheim, Germany; mixture: 70% N_2_O/30% O_2_; vaporisator VIP 3000, Matrix, New York, NY, USA). The body temperature was adjusted to 37°C by a thermostatically controlled warming pad with rectal probe (Fine Science Tools), also after surgery until recovery from anesthesia.

MCAO in sheep was induced as previously described by Nitzsche et al. (2016). Briefly, the subject was anesthetized by an intravenous injection of ketamine (4 mg/kg body weight; Ketamin, Medistar, Holzwicke, Germany), xylazine (0.1 mg/kg body weight; Xylazin, Ceva Sante Animal GmbH, Düsseldorf, Germany) and diazepam (0.2 mg/kg body weight; Temmler Pharma GmbH, Marburg, Germany). During surgery anesthesia was maintained by mechanical ventilation with 2% isoflurane and 40% oxygen (Primus, Dräger AG, Lübeck, Germany). After surgical preparation, the left temporal bone was exposed by incision of the covering skin. Subsequently, the temporal muscle was lifted and trepanation of the underlying bone was performed at 10,000 rpm with a 6 mm barrel burr (microspeed uni, scil animal care company, Viernheim, Germany). Next, the dura mater was incised and the proximal MCA was occluded by electrosurgical coagulation using neurosurgical bipolar forceps (ME 411, KLS Martin, Tuttlingen, Germany). Finally, the dura mater was repositioned, muscles and skin were sutured and the animals were treated with antibiotics (enrofloxacine, 5% Baytril, Bayer AG, Leverkusen, Germany) and analgesics (Butorphanol, Alvegesic 1%; CP-pharm, Burgdorf, Germany) and allowed to wake up after surgery. Two weeks after ischemia onset, sheep were anesthetized again by an intravenous injection of the above mentioned combination of ketamine/xylazine and midazolam (0.2 mg/kg bodyweight; Midazolam, Braun Melsungen, Melsungen, Germany) followed by euthanasia.

Successful induction of stroke in mice and rats was verified by the existence of a relevant neurobehavioral deficit. In detail, during the 24-h observational period, animals had to present at least 2 points on the Menzies score (Menzies et al., 1992), naturally ranging from 0 (no deficit) to 4 (spontaneous contralateral circling). In sheep, animals had to present a visible ischemic lesion in the cerebral magnetic resonance tomography (Biograph mMR, Siemens Healthcare, Erlangen, Germany) that was done at the end of the 2-week observational period.

### Tissue Preparation

Twenty-four hours after induction of ischemia, euthanized mice and rats were transcardially perfused with saline and 4% phosphate-buffered paraformaldehyde. The brains were post-fixed in the same fixative overnight and equilibrated in 30% phosphate-buffered sucrose. Forebrains of rodents were serially cut with a freezing microtome (Leica SM 2000R, Leica Biosystems, Wetzlar, Germany) producing 10 series of 30 μm-thick sections each.

The sheep were euthanized by intravenous administration of a mixture containing embutramide, mebozonium and tetracaine (20 mg/kg, 5 mg/kg and 0.5 mg/kg body weight, respectively; obtained as T61, Intervet Deutschland, Germany), followed by removal of the calvarium. Subsequently, about 10 mm-thick coronal slices were prepared and classified according to a sheep brain atlas (Nitzsche et al., 2015). Each slice was photographed and immersed into 4% buffered formaldehyde for the next 14 days. The slices were then equilibrated in 30% phosphate-buffered sucrose and the immersion-fixed tissue blocs consecutively cut at 40 μm-thickness using a freezing microtome (Microm HM 430, Thermo Fisher Scientific, Waltham, MA, USA). All specimens were collected in 0.1 M Tris-buffered saline (TBS), pH 7.4 containing 0.2% sodium azide and stored at 4°C prior to histochemical labeling.

### Histochemistry

Free-floating sections were rinsed with TBS prior to all histological procedures which commenced by blocking of non-specific binding sites for subsequently applied immunoreagents with 5% normal donkey serum in TBS containing 0.3% Triton X-100 for 1 h. Next, all sections were incubated overnight with mixtures of primary antibodies and biotinylated WFA—diluted in the blocking solution—as listed in Table 1. Subsequently, the sections were washed with TBS and transferred for 1 h into mixtures of appropriate fluorochromated secondary immunoreagents (all at 20 μg/ml TBS-BSA and obtained from Dianova, Hamburg, Germany) according to Table 1. In control experiments, the omission of primary antibodies or biotinylated WFA resulted in the expected absence of cellular labeling. Generally, sections were finally rinsed several times with TBS and briefly in distilled water, mounted onto fluorescence-free glass slides, air-dried and coverslipped with Entellan in toluene (Merck, Darmstadt, Germany).

**Table 1:** Multiple fluorescence labeling.

First primary antibodies	First visualizing immunoreagents	Second primary antibodies	Second viualizing immunoreagents
Immunolabeling combined with the Cy2-staining of WFA-binding sites in mice, rats and sheep*			
rabbit-anti-collagen IV (1:100; Merck Millipore, Billerica, MD, USA)	Cy3-donkey-anti-rabbit IgG	guinea pig-anti-Iba (1:100; Synaptic Systems, Göttingen, Germany)	Cy5-donkey-anti-guinea pg IgG
rabbit-anti-collagen IV (1:100; Merck Millipore)	Cy3-donkey-anti-rabbit IgG	guinea pig-anti-GFAP (1:200; Synaptic Systems)	Cy5-donkey-anti-guinea pig IgG
guinea pig-anti-parvalbumin (1:200; Synaptic Systems)	Cy3-donkey-anti-guinea pig IgG	rabbit-anti-collagen IV (1:70; Merck Millipore)	Cy5-donkey-anti-rabbit IgG
guinea pig-anti-parvalbumin (1:200; Synaptic Systems)	Cy3-donkey-anti-guinea pig IgG	rabbit-anti-aggrecan (1:100; Merck Millipore)	Cy5-donkey-anti-rabbit IgG
Immunolabeling combined with the Cy2-staining of WFA-binding sites in rodents*			
sheep-anti-neurocan (1:200; R&D Systems, Wiesbaden-Nordenstedt, Germany)	Cy3-donkey-anti-goat IgG	rabbit-anti-aggrecan (1:200; Merck Millipore)	Cy5-donkey-anti-rabbit IgG
goat-anti-versican (1:20; R&D Systems)	Cy3-donkey-anti-goat IgG	rabbit-anti-aggrecan (1:100; Merck Millipore)	Cy5-donkey-anti-rabbit IgG
goat-anti-hHAPLN1 (1:100; R&D Systems)	Cy3-donkey-anti-goat IgG	rabbit-anti-aggrecan (1:100; Merck Millipore)	Cy5-donkey-anti-rabbit IgG
rabbit-anti-Kv3.1b (1:1000; Härtig et al., 1999)	Cy3-donkey-anti-rabbit IgG	guinea pig-anti-parvalbumin (1:300; Synaptic Systems)	Cy5-donkey-anti-rabbit IgG
rabbit-anti-Kv3.1b (1:1000)	Cy3-donkey-anti-rabbit IgG	guinea pig-anti-NeuN (1:200; Synaptic Systems)	Cy5-donkey-anti-guinea pig IgG
rabbit-anti-parvalbumin (1:500; Synaptic Systems)	Cy3-donkey-anti-rabbit IgG	guinea pig-anti-NeuN (1:200; Synaptic Systems)	Cy5-donkey-anti-guinea pig IgG

*In general, biotinylated WFA (15 µg/ml; Vector) and mixtures of primary antibodies were concomitantly applied overnight followed by cocktails of Cy2-streptavidin, Cy3- and Cy5-tagged secondary antibodies. All fluorescent immunoreagents were obtained from Dianova (Hamburg, Germany) and used at 20 µg/ml for 1 h. Abbreviations: WFA, *Wisteria floribunda* agglutinin; Iba, ionized calcium binding adapter molecule 1; GFAP, glial fibrillary acidic protein; hHAPLN1, human hyaluronan and proteoglycan link protein (cartilage link protein 1, CRTL); NeuN, neuronal nuclei.

### Microscopy, Image Processing and Semi-Quantification

An Axioplan fluorescence microscope (Zeiss, Germany) was used for the screening of fluorescently labeled brain sections. Images from entire sections and from selected regions at various magnifications were obtained with the microscope Biorevo BZ-9000 (Keyence, Neu-Isenburg, Germany) and the confocal laser-scanning microscope LSM 510 Meta from Zeiss. Panels of micrographs were created with Microsoft PowerPoint (version 2015; Microsoft Corp., Redmond, WA, USA). If necessary, brightness and contrast of micrographs were slightly adjusted without deletion or creation of signals.

Semi-quantifications were performed for collagen IV (Coll IV) and WFA-staining in the ischemia-affected neocortical layers II, III and IV of the somatosensory and parietal cortices, based on images that were captured with the Biorevo BZ-9000 microscope (Keyence). Thereby, three consecutive brain sections with a distance of 300 μm were selected, while the middle section had to demonstrate the most pronounced ischemic lesion involving neocortical areas. Next, analyses involved a forebrain region of about 600 μm in a rostral-occipital direction. In each of the three brain sections, the ischemic region and adjacent non-affected neocortex was subdivided into eight equally spaced regions of interest (ROIs) with a distance of 72 μm between their boundaries. From medial to lateral, the fourth ROI was positioned directly on the sharp border of changed Coll IV-immunoreactivity. For controls, the pattern of the eight ROIs in the ischemic hemisphere was mirrored to the contralateral, non-affected hemisphere, resulting in 16 ROIs in each of the three brain sections per animal. In detail, ROIs were captured using a 40× objective, a field dimension of 181 × 136 μm and a constant exposure time of 1/50 s for Coll IV and 1/55 s for WFA with a 12 bit CCD camera. For quantification of the fluorescence signals, the integrated density—reflecting mean gray values and analyzed area—was measured using ImageJ software (National Institutes of Health, Bethesda, MD, USA) in each ROI.

For all measurements, the threshold was adjusted to the contralateral hemisphere and kept constant at gray values of 1000 for WFA and 1250 for Coll IV to correct for any background intensities relating to non-labeled areas, such as the lumen of cross-sectioned vessels. Thresholds had been adjusted individually for each staining until the background intensity could be nearly excluded without affecting the visualization of targeted structures to prevent adulterations of the applied measurements. We thereby prevented an adulteration of our measurements, since background staining for the relevant markers was lowered in the ischemia-affected tissue. Data were processed by calculating mean values per animal to compare interhemispheric differences for Coll IV and WFA.

### Statistical Analyses

Calculations were performed with the IBM SPSS Statistics package version 24.0 (IBM Corp., New York, NY, USA). After descriptive statistics, the Wilcoxon test was applied to check for statistical significance between both hemispheres. Data are given as means ± standard deviation, unless otherwise indicated. In general, *p* < 0.05 was considered as statistically significant.

## Results

In mice and rats, a Menzies score of at least 2 points was observed during the 24-h observation period, indicating sufficient cerebral ischemia as inclusion criteria for entering the study. In sheep, cerebral infarcts were observed in the temporal lobe and in the striatum via magnetic resonance tomography, while maceration and liquefaction next to brain tissue edema were also macroscopically visible.

### Characterization of PNs by WFA-Staining and Collagen IV-Immunolabeling

**Figure 1:**
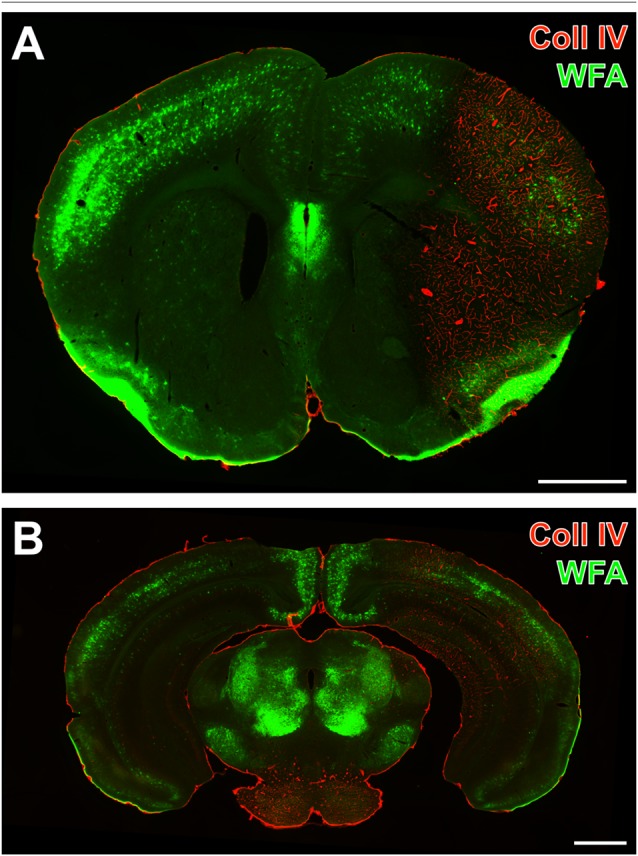
Concomitantly up-regulated collagen IV (Coll IV)-immunoreactivity and decomposed perineuronal nets (PNs) in a mouse 1 day after ischemia onset. Strong Cy3-immunolabeling of Coll IV (red) indicates ischemia-affected regions with a reduced lectin-histochemical *Wisteria floribunda* agglutinin (WFA)-staining (green) in coronal sections from the forebrain comprising striatum and somatosensory cortex **(A)**, as well as the auditory and visual cortex, the caudal hippocampus and the pontine gray matter **(B)** revealed by a Keyence microscope. Scale bars = 1 mm.

**Figure 2:**
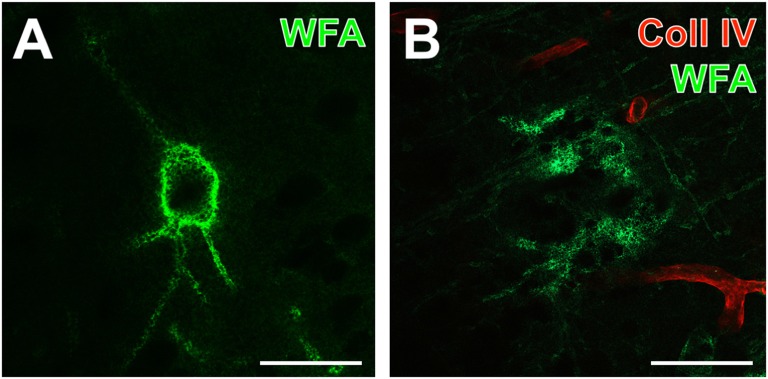
Detailed analysis of up-regulated Coll IV-immunoreactivity and decomposed PNs in a mouse 1 day after ischemia onset. **(A,B)** Confocal laser-scanning micrographs of a highly magnified WFA-positive PN **(A)** in non-affected contralateral somatosensory neocortex devoid of visible Coll IV-immunoreactivity and decomposed net structures **(B)** in the ischemic somatosensory area identifiable by red fluorescent Coll IV-immunoreactivity. Scale bars = 25 μm.

One day after induction of focal cerebral ischemia in mice, we observed opposite alterations for the vessel-associated Coll IV and binding sites for the PN marker WFA, while Coll IV-immunoreactivity became increasingly visible in ischemic areas including the striatum and neocortical structures. A considerable loss of WFA-stainable ECM and decomposed PNs were noted as exemplified by images from whole forebrain sections including affected somatosensory cortex and dorsal striatum (Figure 1A), as well as visual and auditory cortical areas, hippocampal formation and pontine gray matter (Figure 1B). Morphological changes of PN structures became visible at high magnification by confocal laser-scanning microscopy. While Figure 2A shows a representative PN with honeycomb-like meshes ensheathing the whole perikaryon and surrounding the proximal dendrites in a non-ischemic area, Figure 2B presents only WFA-stainable remnants devoid of net-like structures in close vicinity to apparently increased vascular Coll IV-immunoreactivity within the ischemia-affected tissue.

### Semi-Quantification of WFA- and Collagen IV-Staining Following Ischemia

**Figure 3:**
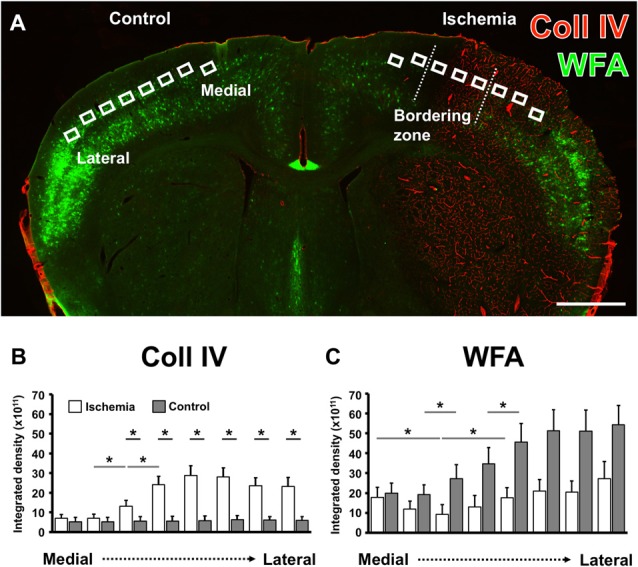
Comparative semi-quantitative analyses addressing immunoreactivity for Coll IV- and WFA-staining in the neocortex of mice. A coronal brain section illustrates the selected regions of interest (ROIs) used for semi-quantitative analysis in the ischemia-affected hemisphere and in the corresponding contralateral area that served as control **(A)**. The semi-quantitative comparison of the Coll IV-immunoreactivity **(B)** and WFA-staining **(C)** between both hemispheres (gray bars indicate the contralateral and white bars the ischemia-affected hemisphere) shows a significant up-regulation of the Coll IV-fluorescence signal that corresponds with a relatively reduced lectin-histochemical signal for WFA alongside from the medial to the lateral part of the neocortex. Bars indicate mean values, and added lines stand for the standard error of mean. Horizontal lines with added significance levels represent inter-hemispheric comparisons (short lines) as well as intra-hemispheric comparisons (long lines). *p < 0.05. Scale bar = 1 mm.

By using eight ROIs on the ischemia-affected hemisphere capturing the ischemic bordering zone, i.e., the transition from the non-affected to the ischemia-affected tissue in direction from medial to lateral, semi-quantitative analyses were added in order to verify the afore described patterns of Coll IV-immunolabeling and WFA-staining. To consider the natural patterns of PN components, the mirrored ROIs on the contralateral hemisphere served as controls (Figure 3A). Thereby, the Coll IV-immunoreactivity on the non-affected hemisphere was found to provide a consistent pattern with nearly equal values for the integrated density from medial to lateral (gray bars in Figure 3B). In contrast, ischemia led to a significant increase of Coll IV-immunoreactivity (white bars in Figure 3B) from the second (7.1 × 10^11^ ± 4.3 × 10^11^) to the third (13.1 × 10^11^ ± 6.7 × 10^11^) and continued to the fourth (24.2 × 10^11^ ± 9.3 × 10^11^) ROI (each *p* = 0.043; Wilcoxon test, *n* = 5). Consequently, direct inter-hemispheric comparison revealed a significant increase of Coll IV-immunoreactivity starting in the ischemic bordering zone and continued towards the most lateral ROI (each *p* = 0.043; Wilcoxon test, *n* = 5). Concerning WFA-binding sites, an ascending increase was observed in the non-affected hemisphere from medial to lateral (gray bars in Figure 3C), representing the natural pattern in neocortical areas. Thereby, a significant increase occurred from the second (19.2 × 10^11^ ± 11.0 × 10^11^) to the third (27.3 × 10^11^ ± 15.8 × 10^11^) as well as between the fourth (34.8 × 10^11^ ± 18.2 × 10^11^) to the fifth (45.7 × 10^11^ ± 20.6 × 10^11^) ROI (each *p* = 0.043; Wilcoxon test, *n* = 5). On the ischemia-affected hemisphere (white bars in Figure 3C), WFA-binding exhibited a shaped course in the bordering zone of ischemia by decreasing significantly from the first (17.7 × 10^11^ ± 11.6 × 10^11^) to the third (9.28 × 10^11^ ± 10.7 × 10^11^), followed by an increase towards the fifth (17.5 × 10^11^ ± 11.4 × 10^11^) ROIs (each *p* = 0.043; Wilcoxon test, *n* = 5). The pattern of WFA-binding sites provided—at least starting at the fourth ROI—a trend towards a diminished relative labeling. The direct inter-hemispheric comparison on the level of each ROI, however, barely missed statistical significance (regions 4–8, each *p* = 0.080; Wilcoxon test, *n* = 5).

### Spatial Relationships between Altered PNs and Cellular NVU Components after Ischemia

**Figure 4:**
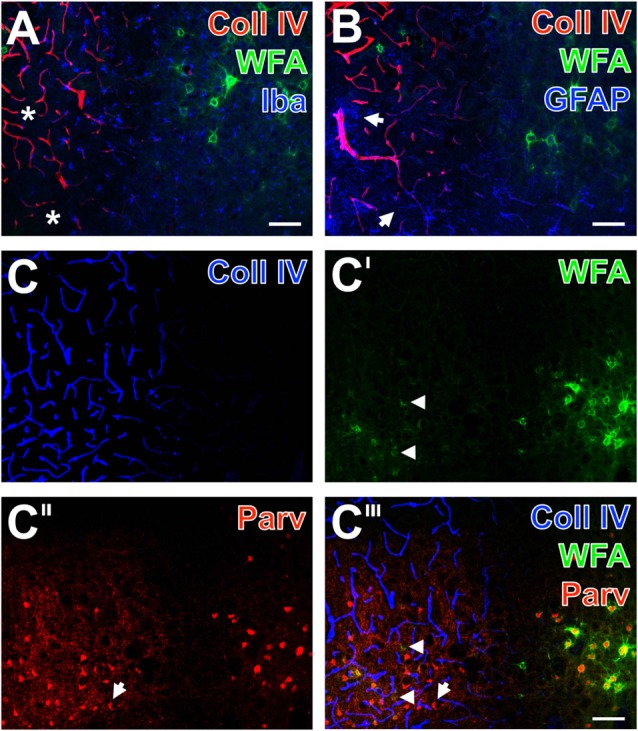
Infarcted mouse: up-regulated Coll IV and decomposed PNs in ischemic neocortex—allocated with microglia/macrophages, activated astroglia and altered parvalbumin-immunolabeling. The ischemic border zone in the somatosensory cortex **(A,B)** exhibits nearly complementary distribution patterns for WFA (green) and strongly up-regulated Coll IV (red) 1 day after induction of ischemia. Additionally, **(A)** displays in the apparently non-affected zone evenly distributed ionized calcium binding adaptor molecule 1 (Iba)-positive microglia (Cy5, color-coded in blue), whereas the infarcted zone is partly devoid of Iba-immunoreactivity (asterisks). In parallel, glial fibrillary acidic protein (GFAP)-immunolabeling (blue) in **(B)** reveals also fragmented astrocytes (arrows) in the damaged tissue. **(C–C‴)** show concomitant staining of Coll IV, WFA and parvalbumin as markers for most neocortical net-bearing neurons. Up-regulated Coll IV-immunoreactivity **(C)** indicates the ischemia-affected tissue, where WFA-stained nets appear decomposed (arrowheads in **C′** and the merged picture **C‴**) or even abolished. Parvalbumin-immunolabeling **(C″,C‴)** visualizes shrunken neurons (exemplified by an arrow) and enhanced neuropil staining in the same area. Here, the overlay **(C‴)** elucidates only a few PNs around parvalbumin-containing neurons. Scale bar in **A,B,C‴** (also valid for **C–C″**) = 75 μm.

**Figure 5:**
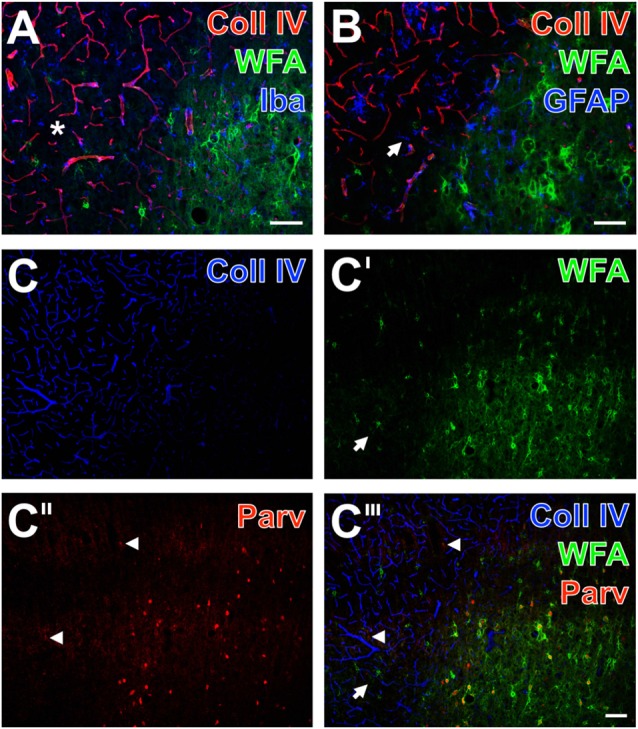
Infarcted rat: up-regulated Coll IV and decomposed PNs in ischemic neocortex—allocated with microglia/macrophages, activated astroglia and altered parvalbumin-immunolabeling. One day after ischemia onset, Coll IV immunostaining (red) appears up-regulated in the parietal cortex with strongly diminished WFA-stained PNs (green) and is considerably lower in the surrounding tissue displaying numerous PNs and neuropil stained for WFA **(A,B)**. Concomitant immunolabeling visualizes predominantly microglia with hardly distinguishable morphology in the differently affected tissue. Additionally, **(A)** shows in the non-affected tissue evenly distributed Iba-positive microglia (Cy5, color-coded in blue), whereas the infarcted zone is partly devoid of Iba-immunoreactivity (asterisk). In parallel, GFAP-immunolabeling (blue) in **(B)** reveals also fragmented astrocytes in the damaged tissue as exemplified by an arrow. **(C–C‴)** Present the concomitant staining of Coll IV, WFA and parvalbumin as marker for most neocortical net-bearing neurons. Up-regulated Coll IV-immunoreactivity **(C)** indicates ischemia-affected tissue, where WFA-stained nets appear decomposed (arrows in **C′** and the merged picture **C‴**) or even abolished. Parvalbumin-immunolabeling **(C″,C‴)** enhanced neuropil staining (arrowheads) in the same area, where the overlay **(C‴)** elucidates only a few PNs around parvalbumin-containing neurons. Scale bar in **A,B** = 75 μm, in **C‴** (also valid for **C–C″**) = 100 μm.

**Figure 6:**
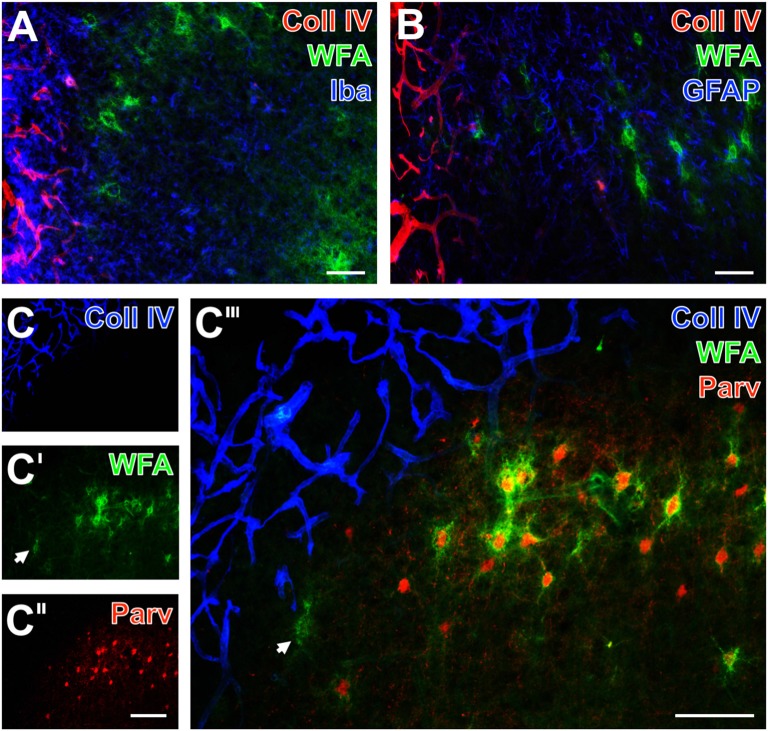
Infarcted sheep: up-regulated Coll IV and decomposed PNs in ischemic neocortex—allocated with microglia/macrophages, activated astroglia and altered parvalbumin-immunolabeling. Nearly complementary staining patterns in the temporal and parietal cortices are visible for strong Coll IV-immunoreactivity restricted to ischemia-affected regions and WFA-stained PNs 14 days after ischemia onset **(A,B,C‴)**. Simultaneous detection of Iba in (**A**; blue) reveals a strongly up-regulated immunosignal for microglia/macrophages in the Coll IV-stained region vs. ameboid and ramified microglial cells in close vicinity to PNs. Concomitant staining of GFAP, WFA and Coll IV reveals activated astrocytes (blue) predominantly around the heavily Coll IV-stained region forming a glia scar-like structure **(B)**. Triple staining of Coll IV (blue), PNs (green) and parvalbumin (red) at the ischemic border detects Coll IV exclusively within the affected tissue **(C)** which displays only remnants of PNs (arrow in **C′**), whereas the healthy tissue contains numerous WFA-stained PNs **(C′,C‴)** around parvalbumin-containing neurons **(C″,C‴)**. The overlay **(C‴)** shows nearly complementary staining patterns for Coll IV and the both other markers. Scale bars in **A,B** = 75 μm, in **C″** (also valid for **C,C′**) = 150 μm, in **C‴** = 100 μm.

By focusing on the relationship between PNs and vascular alterations as indicated by an altered Coll IV-immunoreactivity and glial NVU components, additional qualitative data were obtained from multiple-labeled forebrain sections of rodents 1 day after ischemia onset and from sheep 14 days after induction of ischemia. Additionally, net-associated parvalbumin-containing neurons were visualized to explore characteristics in the neocortical ischemic border zone of mice (Figure 4), rats (Figure 5) and sheep (Figure 6). Thereby, nearly complementary distribution patterns for WFA and massively enhanced Coll IV-immunoreactivity was found in all three species investigated (Figures 4A, 5A, 6A,A′). Remarkably, the infarcted zone of rodents was partly devoid of microglia immunoreactive for the ionized calcium-binding adaptor molecule 1 (Iba; asterisks in Figures 4A, 5A). However, in the representative image from the ischemic border zone of the sheep 14 days after infarction (Figure 6A), strongly Iba-labeled microglia/macrophages can be observed in the Coll IV-stained region, contrasted by ameboid and ramified microglial cells in vicinity to PNs.

**Figure 7:**
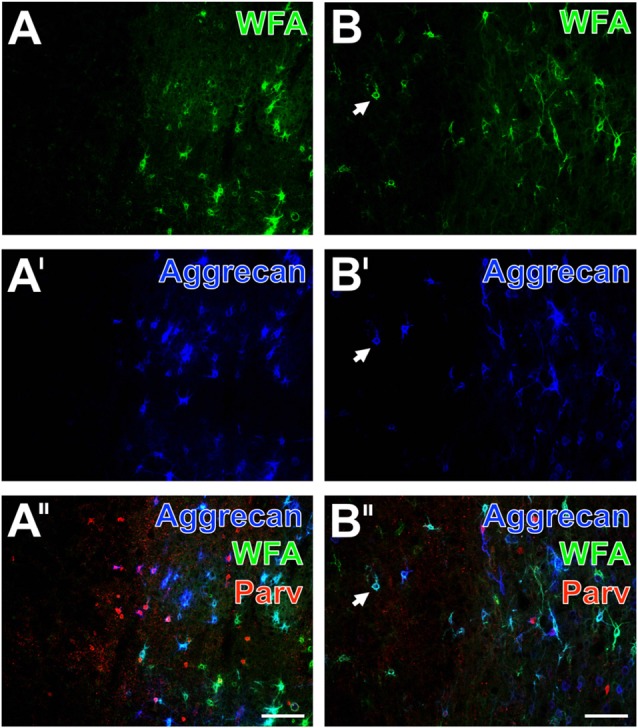
Decomposition of WFA- and aggrecan-positive nets allocated with altered parvalbumin-containing neurons in the neocortex of mouse and rat. In a representative mouse section, the ischemic somatosensory cortex with lowered neuropil staining is devoid of lectin-histochemical stained PNs (**A**, green) and aggrecan-immunopositive nets (**A′**, color-coded in blue). The merge of staining patterns **(A″)** for parvalbumin (red) and PNs reveals that the outer rim of the affected region still displays parvalbumin-immunopositive cells, but also neuropil which appears coarser than in the healthy tissue. Additionally, the overlay visualizes a large portion of pink-appearing nets positive both for WFA and aggrecan as well as allocated parvalbumin-immunoreactive cells devoid of PNs. In a typical rat section, the ischemic border zone in the frontal cortex displays only some remaining nets stainable by WFA (**B**, green) and anti-aggrecan (**B′**, blue) exemplified by arrows. Concomitantly, the parvalbumin-immunolabeling **(B″)** is severely affected and the overlay also displays numerous PNs stainable both by WFA and anti-aggrecan. Scale bars in **A″,B″** (also valid for all other micrographs) = 100 μm.

Additional triple fluorescence labeling of Coll IV, WFA and glial fibrillary acidic protein (GFAP) demonstrated in the affected tissue from mice and rats also fragmented astrocytes at 1 day after ischemia onset (Figures 4B, 5B). In sheep, activated astrocytes became predominantly visible around the heavily Coll IV-stained region and formed a glia scar-like structure (Figure 6B). Analyses focusing on the spatial relationship to parvalbumin—as marker for most neocortical net-bearing neurons—revealed in all three models the expected up-regulated Coll IV-immunoreactivity (Figures 4C, 5C, 6C), delineating ischemia-affected tissue, whereas in the same area WFA-stained nets appeared decomposed (Figures 4C′, 6C′) or were even abolished (Figure 5C′). Parvalbumin-immunolabeling (Figures 4C″, 5C″, 6C″) detected shrunken cells and especially in the rat (Figure 5C″) combined with enhanced labeling of neuropil in the same area. In the ischemia-affected zone, merged staining patterns revealed only a few PNs around parvalbumin-containing neurons (Figure 4C‴) or even the absence of such net-enwrapped neurons (Figures 5C‴, 6C‴) while on the contrary, the healthy tissue in all models contained numerous WFA-stained PNs around parvalbumin-containing neurons. Strikingly, the overlay demonstrated nearly complementary staining patterns for Coll IV on the one hand and WFA and parvalbumin on the other hand. To address the CSPG aggrecan as a further PN constitute, triple fluorescence staining combined with the detection of WFA and parvalbumin was performed in neocortices of mice (Figures 7A–A″) and rats (Figures 7B–B″). Thereby, mice displayed a neocortical ischemia-affected area with lowered lectin-staining of the neuropil and devoid of PNs detected by WFA (Figure 7A) and aggrecan (Figure 7A′). After merging of staining patterns for PNs and parvalbumin, the calcium-binding protein was also observed in affected tissue, i.e., diminished in cells and in the neuropil with a coarser appearance than in healthy tissue (Figure 7A″). The overlay further visualized co-localized WFA-binding sites and aggrecan-immunoreactivity as well as parvalbumin-containing cells devoid of PNs. In parallel, neocortical ischemia-affected rat tissue showed a few remaining PNs recognized by WFA (Figure 7B, green) and anti-aggrecan (Figure 7B′, blue), each exemplified by an arrow. Concomitantly, parvalbumin-immunolabeling (Figure 7B″) was severely affected, while only a few apparently intact PNs surrounded cells with detectable calcium-binding protein remained and the neuropil appeared again coarse. As expected, the merge of staining patterns also visualized PNs double-labeled by WFA and anti-aggrecan (Figure 7B″).

### Combined Detection of Distinct Net Components after Ischemia

To investigate further ischemia-affected net constituents in rodents, the immunodetection of neurocan, versican and the cartilage link protein (CRTL) was combined with double labeling of WFA and aggrecan.

**Figure 8:**
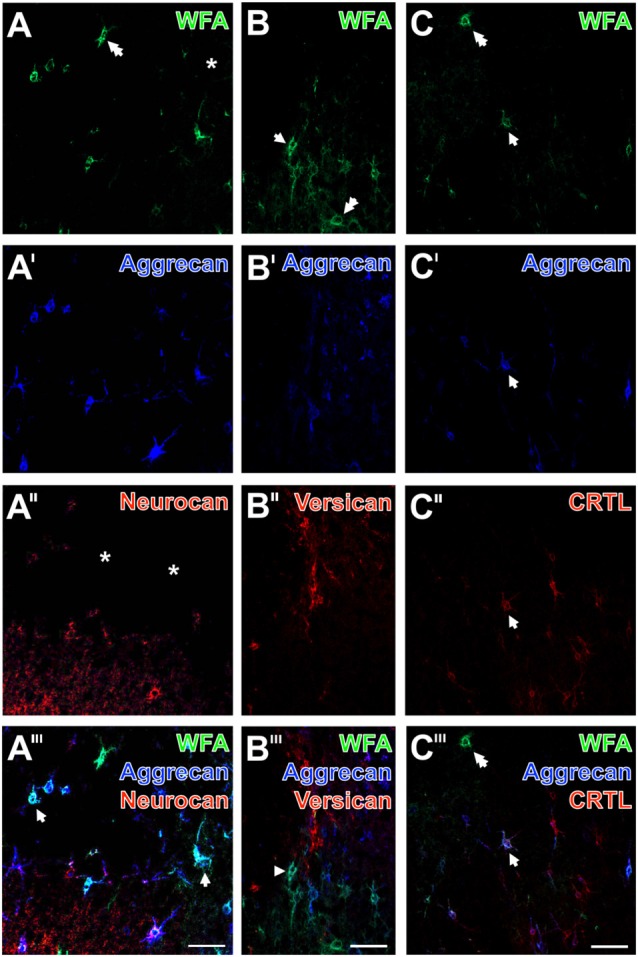
Ischemia-affected net constituents neurocan, versican and cartilage link protein (CRTL) vs. decomposed WFA-binding and aggrecan-containing PNs in rat neocortex. One day after ischemia onset, confocal laser-scanning shows that WFA-stained extracellular matrix (ECM; **A–C**; green) and aggrecan-immunolabeling (**A′–C′**; blue) are similarly abolished in ischemia-affected frontal cortex. Remarkably, some WFA-positive nets are devoid of a co-staining (two-headed arrow in **A,C**). The combined detection with neurocan (**A″**, red) reveals not only PNs but also a neuropil staining which is diminished in the damaged area (asterisk). Concomitant immunolabeling of versican (**B″**, red) shows several decomposed nets in close vicinity to the strongly affected tissue, whereas anti-CRTL (**C″**, red) stains in the presented case apparently more PNs than WFA and anti-aggrecan. The merged staining patterns **(A‴–C‴)** visualize triple-stained nets (arrows), numerous WFA/aggrecan co-labeled nets (arrowhead) and nets mono-labeled by WFA (two-headed arrow). Scale bars in **A‴–C‴** (also valid for all other micrographs) = 75 μm.

Representative mouse sections displayed that the ECM stainable by WFA (Figures 8A–C; green) and aggrecan (Figures 8A′–C′; blue) were similarly abolished in the ischemia-affected area, which was also largely devoid of neurocan-immunolabeling (Figures 8A″,A‴). Notably, the apparently healthy tissue displayed a strong neuropil staining for neurocan. In parallel, immunolabeling of versican revealed several damaged PNs in close vicinity to the strongly affected tissue (Figure 8B″), whereas the detection of CRTL visualized in the presented area more PNs than WFA and anti-aggrecan (Figures 8C″,C‴). The overlay (Figures 8A‴,B‴,C‴) elucidated triple-stained nets (arrows), numerous WFA/aggrecan co-labeled nets (arrowheads) and nets mono-labeled by WFA (two-headed arrow).

**Figure 9:**
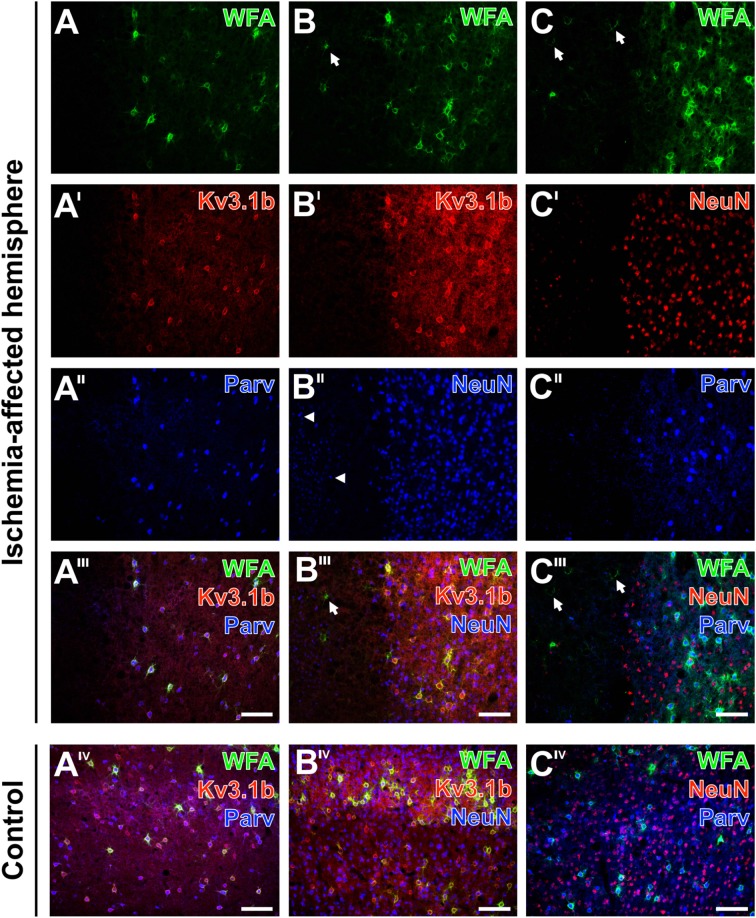
Alterations of parvalbumin, Kv3.1b and neuronal nuclei (NeuN) in neurons surrounded by ischemia-affected, WFA-stainable nets in mouse neocortex. One day after stroke induction PNs at the ischemic border zone in the neocortex are revealed by WFA-staining (green) in combination with the immunolabeling of **(A–A‴)** Kv3.1b + parvalbumin, **(B–B‴)** Kv3.1b + NeuN and **(C–C‴)** NeuN + parvalbumin. Evenly distributed PNs in the apparently healthy tissue contrast to the ischemia-affected area **(A)** devoid of WFA-labeled structures or **(B,C)** with only some remnants of nets (arrows in **B,B‴,C,C‴**). Kv3.1b and parvalbumin are co-localized in all detected neurons **(A′–A‴)** which results in the pink appearance of neurons co-expressing both markers **(A‴)**. While Kv3.1b and NeuN-immunolabeling appear both unaltered in the sharply delineated healthy tissue, the ischemia-affected region additionally contains structures with diminished NeuN-immunosignals (arrowheads in **B″**). Additionally, NeuN-staining **(C′)** is also less affected in comparison to parvalbumin-immunoreactivity which remains restricted to coarse neuropil staining in the injured tissue **(C″)**. Merged images show all three marker combinations both in the ischemia-affected neocortex **(A‴–C‴)** and in the same neuroanatomical area on the contralateral, non-affected side **(A^IV^–C^IV^)**. Scale bars **A‴,A^IV^,B‴,B^IV^,C‴,C^IV^** (also valid for all other micrographs) = 100 μm.

The ischemic border in figures devoid of Coll IV-immunolabeling (Figures 8, 9) was localized by comparison of the presented sections with Coll IV-stained consecutive sections or by a lowered background staining typically seen in the ischemic tissue.

### Different Affection of Net-Bearing Neurons after Ischemia

The distribution of WFA-binding sites in the PNs was compared with alterations of immunoreactivities for parvalbumin, Kv3.1b—which are both known as markers for fast-firing GABAergic neurons—and the “pan-neuronal” marker neuronal nuclei (NeuN) in neocortical neurons of mice (Figure 9) and rats (data not shown).

Thereby, lectin-positive PNs at the neocortical ischemic border were concomitantly revealed with Kv3.1b + parvalbumin (Figures 9A–A‴), Kv3.1b + NeuN (Figures 9B–B‴) and NeuN + parvalbumin (Figures 9C–C‴). In contrast to the rather evenly distributed PNs in the healthy tissue, the ischemia-affected areas were either devoid of WFA-labeled structures (Figure 9A) or exhibited only some remnants of nets (Figures 9B,C). In general, Kv3.1b (Figure 9A′) and parvalbumin (Figure 9A″) were co-localized, and in the overlay neurons co-expressing both markers appeared pink (Figure 9A‴). Notably, cells displaying only one of both proteins were not observed. On the contrary, the ischemia-affected region contained structures with diminished NeuN-immunoreactivity (Figures 9B″,C′) without an immunosignal for Kv3.1b (Figure 9B‴), and only parvalbumin-stained coarse neuropil as well as a few remnants of shrunken neurons (Figure 9C″). In contrast, triple fluorescence labeling in the same areas on the contralateral, non-affected hemisphere displayed homogeneous patterns for all three applied combinations of markers devoid of any cellular affection (Figures 9A^IV^–C^IV^).

## Discussion

The present study aimed to explore ischemia-associated histopathological alterations of PNs and their spatial relationships to vascular, glial and neuronal elements to improve the currently limited knowledge on complex interactions between the cellular components of the NVU and adjacent extracellular structures. These results might help to develop new treatments in acute ischemic stroke centered on targeting the integrity of the NVU with associated ECM components.

We employed a variety of triple fluorescence labeling techniques including a variety of primary and secondary antibodies as well as lectin-based staining, which allowed the detection of simultaneous changes of vessels, glia, neurons and ECM components in the NVU. Since the importance on the selection of an appropriated stroke model is highlighted by the still existing translational roadblock (Endres et al., 2008; Fisher et al., 2009), in this study efforts were made to consider translational aspects by using three stroke models with clinical implications. Thereby, the filament model in mice (Longa et al., 1989) allowed non-invasive induction of focal cerebral ischemia and led to reproducible infarction in the territory of the middle cerebral artery (Longa et al., 1989; Durukan and Tatlisumak, 2007; Engel et al., 2011), enabling robust quantifications on cellular reactions. Applying this model, our group already described ischemia-associated changes in endothelial cells, up-regulation of Coll IV in basal membranes and loss of aquaporin 4 in astrocytic endfeet 24 h after ischemia induction (Hawkes et al., 2013). Furthermore, we recently observed alterations on cytoskeletal elements as visualized by tau-immunoreactivity and abolished PNs in the nucleus reticularis thalami due to permanent focal cerebral ischemia (Härtig et al., 2016; Michalski et al., 2016). The applied thromboembolic rat model is currently considered as the most patient-near animal model, since the ischemic event is strictly caused by an embolus in excellent accordance to the human situation where cardio-embolism due to atrial fibrillation and arterial embolism—based on arteriosclerosis or carotid stenosis—accounts for the most frequent causes of stroke (Durukan and Tatlisumak, 2007; Young et al., 2007; Sommer, 2017). According to current recommendations for improving experimental stroke research (Stroke Therapy Academic Industry Roundtable (STAIR), 1999; Fisher et al., 2009), an implementation of gyrencephalic, large animal models in preclinical stroke research is desirable. Therefore, we analyzed not only two different rodent models, but also a sheep model of focal cerebral ischemia (Boltze et al., 2008). As an advantage, the sheep model shares anatomical and pathohistological similarities to the human situation (Nitzsche et al., 2015).

### Stroke-Induced Alterations of PNs in Association with NVU Constituents

Qualitative analyses in our study implicated the concomitant degradation of PNs and up-regulation of Coll IV in the ischemia-affected neocortices of mice, rats and sheep as the main finding of the present study. The added semi-quantification of Coll IV confirmed this observation in terms of a significantly up-regulated immunoreactivity in ischemic areas, confirming our previous findings using other mouse strains including a transgenic model with Alzheimer-like alterations and aged animals (Hawkes et al., 2013). Concerning the underlying causes, it remains to be clarified whether the increase in Coll IV-immunoreactivity corresponds to a reactive feature with the aim to stabilize vascular elements against the ischemic stimulus, or as kind of a degradation which leads to an increased level of antigens detectable by the applied immunohistochemical method. However, as matrix metalloproteinases have been considered to influence the vascular integrity and the ischemic stimulus is known to promote the production of matrix metalloproteinases (del Zoppo, 2009, 2010), the robustly observed up-regulation of Coll IV across the three animal models might be seen in the context of these mediators.

The present study revealed novel data on WFA-staining patterns at the border zone of the ischemia-affected neocortex 24 h after experimental stroke. Thereby, semi-quantification provided evidence for a shaped course with a significant drop directly at the border zone towards the ischemia-caused cellular alterations including clear cut up-regulation of Coll IV. Furthermore, the WFA-staining was relatively diminished in the area of ischemia. In detail, we were able to show an increase of WFA-staining in the medial to lateral direction at the non-ischemic hemisphere, which is assumed to represent the natural course in this neocortical region. In contrast, on the contralateral, ischemia-affected hemisphere such increased WFA-staining is lacking.

Additional novel qualitative data are the detection of reduced and structurally altered net components neurocan, versican and the CRTL. In line with previously reported data on the nucleus reticularis thalami (Härtig et al., 2016), the present study provides evidence for diminished neocortical PN markers in association with net-bearing, fast-firing GABAergic neurons such as parvalbumin and the potassium channel subunit Kv3.1b.

Immediately after stroke, matrix metalloproteinases are known to become dysregulated (Heo et al., 1999) and associated with acute neurovascular disruption. However, the same enzymes later contribute to beneficial mechanisms of neurovascular remodeling (Lo, 2008; Rosell and Lo, 2008; del Zoppo, 2010), which makes it difficult to interact pharmacologically in a consistent direction. Further candidates for the stroke-induced degradation of net components are aggrecanases, e.g., disintegrin and metalloproteinase with thrompospondin motifs (ADAMTS-1, 4, 8 and 15) and neprilysin (Cross et al., 2006; Lemarchant et al., 2013; Levy et al., 2015; Rossier et al., 2015). Interestingly, such enzymes were observed in net-ensheathed parvalbumin-immunoreactive neurons (Rossier et al., 2015). After focal cortical ischemia in rats, WFA- binding sites were found to be reduced in the demarcation zone at different time points between 24 h and 60 days after photothrombosis (Bidmon et al., 1998). Based on WFA staining, abolished and decomposed PNs were also reported for other animal models of experimentally induced stroke in neocortices of rats (Hobohm et al., 2005; Karetko-Sysa et al., 2011; Madinier et al., 2014; Quattromani et al., 2017). In line with these findings, the present study shows erased WFA staining in the neocortices from infarcted mice, rats and sheep extending own data on the affected ECM in the nucleus reticularis thalami of mice (Härtig et al., 2016).

Among the addressed net components, we detected strongly degraded aggrecan known as predominant CSPG in PNs occurring in several isoforms (Matthews et al., 2002; Morawski et al., 2012a) with differential distribution as shown for the human cerebral cortex (Virgintino et al., 2009). The fluorescence labeling also verified the occurrence of PNs double-positive for WFA and aggrecan or only for one of both markers. This finding is in line with the recently reported heterogeneity of PNs in the mouse hippocampus (Yamada and Jinno, 2017). The removal of their coatings might allow formerly net-enwrapped parvalbumin-containing neurons to re-open a more plastic state (Hensch, 2014). On the contrary, the loss of inhibitory neurons immunoreactive for parvalbumin and Kv3.1b might impair the GABAergic wiring in the cortical microcircuitry (Kubota, 2014).

### Differently Altered PNs in Further Brain Disorders

Decomposed PNs are not restricted to stroke, as they were also demonstrated for other neurological disorders (Soleman et al., 2012). As for instance, in schizophrenia abnormalities CSPG markers such as WFA, aggrecan and chondroitin-6-sulfates in the ECM and associated glia were demonstrated in the amygdala and entorhinal cortex of cases with schizophrenia (Pantazopoulos et al., 2010). Based on WFA-staining of tissue from 86 *post mortem* human brains a strong decrease of PNs in the prefrontal cortical layers III and V contributes to the prefrontal cortex dysfunction in this disease (Mauney et al., 2013). Therefore, Berretta et al. (2015) discussed that the physiological remodeling of the ECM is disrupted and, in turn, contributes to the dysfunction of GABAergic neurons in schizophrenic patients (Berretta et al., 2015). Impaired PNs and remodeling of the ECM were also repeatedly reported for animal models of epilepsy (Dityatev, 2010; McRae et al., 2012; Rankin-Gee et al., 2015).

Notably, autoptic cases with bipolar disorder displayed in the amygdala and in the entorhinal cortex only negligible changes of CSPGs and only modest decrease of PN markers (Pantazopoulos et al., 2010, 2015). Unaltered PNs were also reported for an established animal model of β-amyloidosis in Alzheimer’s disease (AD), Tg2576 mice with cortical age-dependent vascular and parenchymal deposits of β-amyloid peptides (Morawski et al., 2010). Subsequently, Morawski et al. (2012b) extended these findings by comprehensive data on PNs in autoptic tissues from AD patients confirming the weak changes in PN structures during this neurodegenerative disease. By contrast, Suttkus et al. (2016a) found that the neuronal ECM restricts distribution and internalization of aggregated tau. Interestingly, the vast majority of cortical net-bearing neurons showed no tau hyperphosphorylation in numerous cortical autoptic samples (Brückner et al., 1999), and the neocortex of aged bisons displayed aberrantly phosphorylated tau exclusively in cells devoid of PNs (Härtig et al., 2001).

Apart from the preservation, an enhancement of net components was reported for addiction in experimental models. Repeated binge drinking caused an increase of PNs in the insular cortex of mice (Chen et al., 2015). Changes of brain ECM molecules resulting from addiction to opiates, psychostimulants and alcohol are contrasting to alterations in schizophrenia and mood disorders (Lubbers et al., 2014; Slaker et al., 2016). A single cocaine injection into the prefrontal cortex caused a decreased density of PNs, whereas five daily cocaine injections resulted in an increased density of PNs and lowered plasticity (Sorg et al., 2016).

### Experimental Treatment of PNs

An established tool for the remodeling the ECM and especially PNs *in vivo* and even under pathological conditions are bacterial glycosaminoglycan-degrading enzymes such as chondroitinase ABC (ChABC) as reviewed by Burnside and Bradbury (2014). Injections of ChABC into the rat frontal or occipital cortex resulted in the erasure of PNs within 1 day followed by the significant re-establishment of matrix components only after 4 weeks and the rebuilding of PNs only after 5 months (Brückner et al., 1998).

Following experimentally induced stroke in aged rats, the timely fine-tuned delayed treatment with ChABC promoted functional recovery and neuroplasticity, but did not protect the peri-infarct region (Soleman et al., 2012). Notably, the ChABC-induced removal of negatively charged ECM components such as glycosaminoglycans resulted in enhanced calcium diffusion within brain tissue which influences synaptic transmission, neuronal excitability and other physiological processes (Hrabětová et al., 2009). After thalamic stroke in hypertensive rats, the continuous intra-infarct infusion of ChABC rescued neuronal loss and enhanced levels of synaptophysin and growth-associated protein 43 indicating augmented axonal growth as well as synaptic plasticity to finally ensure overall neuronal function (Chen et al., 2014). Following cortical photothrombotic stroke in rats, enriched housing after ischemia induction reduced PNs, modulated activity and mRNA expression of ECM proteases and their inhibitors, accompanied by enhanced recovery of limb placement ability (Madinier et al., 2014; Quattromani et al., 2017).

Remarkably, ChABC injections into perirhinal cortices from transgenic mice with tau pathology restored even the object recognition memory of these animals (Yang et al., 2015).

Moreover, the ChABC-induced removal of PNs in the medial prefrontal cortex impaired acquisition and reconsolidation of conditioned place preference memory after its impairment by cocaine (Slaker et al., 2015).

### Methodical Considerations

The present study has some limitations. First, despite the use of a broad spectrum of antibodies, a comparable immunolabeling of diverse cellular and ECM structures across the three animal species was in some parts hampered because of some differences in the post-ischemic tissue processing including fixation. In detail, sheep brains were not perfusion-fixed, which was due to the experimental constraints of the underlying sheep studies. Notably, it was possible to carry out immersion fixation of about 1 cm-thick coronal forebrain slices from sheep. Consequently, frozen tissue sections from these blocs allowed for the detection of PNs and multiple vascular, glial and neuronal markers. Notably, several presented staining methods were well applicable in all three animal species analyzed. Thereby, the immunohistochemical toolbox for studies of sheep brain tissue was considerably extended. However, the amount of available sheep and rat tissue sections was restricted in this study, which allowed only qualitative investigations in these species. At least semi-quantifications were feasible in mice based on *n* = 5. Future studies are requested to re-produce the current quantitative findings in larger series of rats and sheep.

It is also noteworthy that experiments were limited to a single time point (i.e., 24 h after ischemia induction in rodents, and 14 days after ischemia induction in sheep). Given the fact that post-stroke reactions were described in a multi-phasic fashion (Dirnagl et al., 1999), additional work is necessary to explore the longitudinal course of addressed markers. This approach would also allow to gain more data on the underlying mechanisms of the observed histochemical alterations in terms of a primary consequence of the ischemic stimulus or a secondary effect driven by the ischemic affection of neighboring cellular structures.

### Outlook

This study confirmed that the ECM and in particular PNs are an important component of the NVU, which critically impacts on proper neuronal function. Therefore, novel insights into the spatial and functional relationship of the ECM and the cellular, especially vascular components of the NVU might help to elaborate additional strategies for an effective neuroprotection. In this context, future studies should not only address the acute phase of ischemic stroke to alleviate cellular damage due to the critical impairment of blood supply (Dirnagl et al., 1999), but further be aimed at later, chronic stages after stroke to improve reorganization and neuronal plasticity (Pekna et al., 2012).

## Author Contributions

WH and DM designed the study and wrote the manuscript. Animal experiments with rodents were carried out by DM and MK, while the experiments with sheep were conducted by HB and BN. Histochemistry and imaging were performed by WH, BM, SAleithe and SAltmann. Semi-quantitative analyses were done by BM, SAleithe, MK and DM. The final figures were generated by DM. All authors edited the manuscript.

## Conflict of Interest Statement

The authors declare that the research was conducted in the absence of any commercial or financial relationships that could be construed as a potential conflict of interest.

## Abbreviations

AD: Alzheimer’s disease
CSPG: chondroitin sulfate proteoglycan
ECM: extracellular matrix
Iba: Ionized calcium binding adaptor molecule 1
pMCAO: permanent middle cerebral artery occlusion
PNs: perineuronal nets
ROI: region of interest
rtPA: recombinant tissue plasminogen activator
TBS: Tris-buffered saline
WFA: *Wisteria floribunda* agglutinin.
